# 2675. Concurrent HIV and Sexually Transmitted Infections in People with Mpox in Guatemala City

**DOI:** 10.1093/ofid/ofad500.2286

**Published:** 2023-11-27

**Authors:** Hugo Marroquin, Alvaro J Mazariegos Farfán, Rodolfo Pinzón, Johanna Samayoa

**Affiliations:** Hospital Roosevelt, Guatemala City, Sacatepequez, Guatemala; Hospital Roosevelt, Guatemala City, Sacatepequez, Guatemala; Hospital Roosevelt, Guatemala City, Sacatepequez, Guatemala; Hospital Roosevelt, Guatemala City, Sacatepequez, Guatemala

## Abstract

**Background:**

High rates of HIV and sexually transmitted infections (STIs) among people with mpox have been reported. Guatemala has the highest number of mpox cases reported in Central America. Data on this topic in the region is lacking. We report the prevalence of STIs and HIV in people evaluated for mpox at Hospital Roosevelt in Guatemala City.

**Methods:**

We conducted a cross-sectional analysis of confirmed mpox cases with outpatient care at our institution between July 2022 and January 2023. Sociodemographic and clinical data was retrospectively collected. This analysis includes new diagnoses of HIV, syphilis, chlamydia, gonorrhea, mycoplasma genitalium infection and herpes virus simplex (HSV) infection. Diagnoses were confirmed by serology or nucleic acid amplification tests. Rates of HIV/STIs were assessed and association with sociodemographic, behavioral and clinical characteristics was analyzed through non-parametric statistical tests.

**Results:**

Sixty participants with mpox were included. All cis-gender male, 34 (56.6%) younger than 30 years old, 52 (86.6%) were residents of Guatemala City, 57 (95%) men who have sex with men (MSM), 51 (85%) had above secondary education, 36 (60%) with known HIV-positive status. Recent participation in group sex was reported in 9 (15%) participants and inconsistent or no condom use in 49 (81.6%). Among participants with known HIV-positive status 34 (94.4%) had CD4 count > 200/µL and 32 (88.9%) had a HIV viral load < 200 copies/ml. Two previously undiagnosed HIV infections were detected. Overall, 27 new STIs were found in 20 (33.3%) participants (5 had more than one STI). Comparisons between participants with concurrent STIs and those without are shown in Table 1. Nine cases of syphilis were detected. Eighteen STIs involved mucous membranes (oropharyngeal, genital or anorectal). Rates by site are shown in Table 2.

**Figure:**
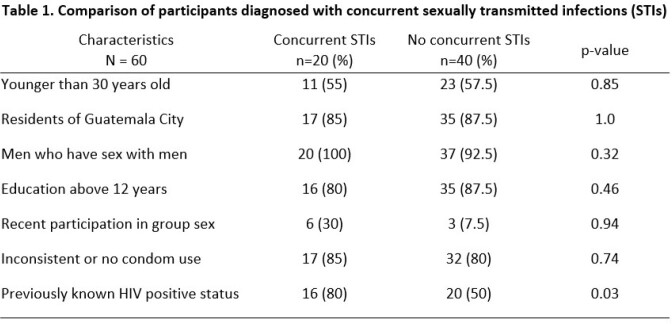

**Figure:**
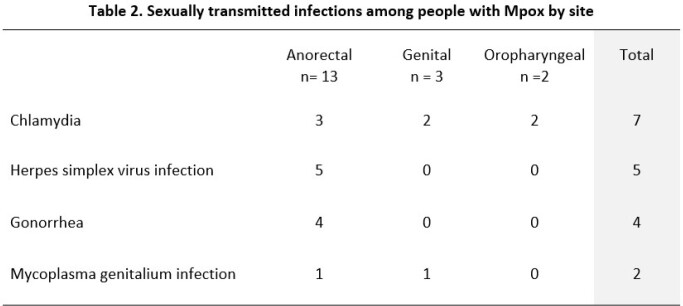

**Conclusion:**

At least one STI was detected in a third of the mpox cases with syphilis being the most frequent. Almost half of STIs cases involved the anorectal site. People with previously known HIV-positive status had a significantly higher rate of STIs. HIV/STI care is key to an appropriate mpox management.

**Disclosures:**

**All Authors**: No reported disclosures

